# Pollinator‐mediated plant coexistence requires high levels of pollinator specialization

**DOI:** 10.1002/ece3.10349

**Published:** 2023-08-01

**Authors:** Bethanne Bruninga‐Socolar, Jacob B. Socolar, Sabine Konzmann, Klaus Lunau

**Affiliations:** ^1^ Biology Department Albright College Reading Pennsylvania USA; ^2^ Natural Capital Exchange San Francisco California USA; ^3^ Universität Düsseldorf Düsseldorf Germany

**Keywords:** bumblebees, foraging behavior, intraspecific variation, mutualism, pollen limitation

## Abstract

In pollen‐limited plant communities, the foraging behavior of pollinators might mediate coexistence and competitive exclusion of plant species by determining which plants receive conspecific pollen. A key question is whether realistic pollinator foraging behavior promotes coexistence or exclusion of plant species. We use a simulation model to understand how pollinator foraging behavior impacts the coexistence dynamics of pollen‐limited plants. To determine whether pollinators are likely to provide a biologically important coexistence mechanism, we compare our results to bee foraging data from the literature and from a novel experimental analysis. Model results indicate that strong specialization at the level of individual foraging paths is required to promote coexistence. However, few empirical studies have robustly quantified within‐bout specialization. Species‐level data suggest that foraging behavior is sufficient to permit pollinator‐mediated coexistence in species‐poor plant communities and possibly in diverse communities where congeneric plants co‐occur. Our experiments using bumblebees show that individual‐level specialization does exist, but not at levels sufficient to substantially impact coexistence dynamics. The literature on specialization within natural foraging paths suffers from key limitations, but overall suggests that pollinator‐mediated coexistence should be rare in diverse plant communities.

## Graphical abstract

A key question for understanding plant‐pollinator interactions is whether realistic pollinator foraging behavior promotes coexistence or exclusion of plant species. We use a simulation model to understand how pollinator foraging behavior impacts the coexistence dynamics of pollen‐limited plants. Model results indicate that strong specialization at the level of individual foraging paths is required to promote coexistence.

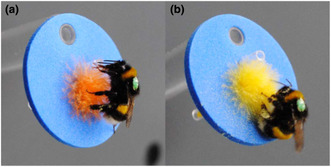

## 1 INTRODUCTION

Two properties of pollinator biology pose challenges to plant species coexistence, particularly the persistence of rare plant species in species‐rich communities. First, pollen is transferred quickly, so successful plant reproduction depends on sequential or nearly sequential visits to conspecific plants (Karron et al., 2009; Richards et al., 2009), that is, flower constancy (Waser, 1986). The sequence of plants visited will depend on the spatial arrangement of plants and pollinator preferences for the plant species in the local environment (Bruninga‐Socolar et al., 2022; Saleh & Chittka, 2007). In the context of dietary generalist pollinators, the frequency of pollination events to a particular plant species does not scale linearly with plant relative abundance. Instead, if pollinator visits are independent, then the probability that a pair of visits will be sequential to the same plant species is the square of the plant species' relative abundance. Thus, pollination by generalist pollinators should tend to exclude rare plants from the community via a positive feedback loop.

Second, foraging pollinators (i.e., bees) tend to preferentially visit common plant species (Augspurger, 1980; Bruninga‐Socolar et al., 2016; Bruninga‐Socolar & Branam, 2022; Dauber et al., 2010; Ghazoul, 2006; Kunin, 1997a; Levin & Anderson, 1970; Moeller, 2004; Podolsky, 1992). This tendency coupled with the dependence of plant reproduction on sequential visits by bees suggests that successful pollination of rare plant species by generalist pollinators should be infrequent, and pollen limitation should exclude rare plant species from the community (Ferrière et al., 2004). Yet diverse communities of bee‐pollinated plants are ubiquitous in nature, and some of these communities are pollen‐limited at least some of the time (Ashman et al., 2004; Knight et al., 2005). How do rare plants find interaction partners and persist in such communities?

For rare species in pollen‐limited communities, adequate pollination requires some form of preferential visitation within foraging paths (Benadi & Gegear, 2018). This preferential visitation might take the form of specialized interactions with pollinator species (Benadi, 2015; Valdovinos et al., 2013), spatial clumping among conspecific plants (Bruninga‐Socolar et al., 2022), or specialization within generalist pollinator species at the level of individuals or even of single foraging paths (Brosi & Briggs, 2013). The potential contribution of individual‐level foraging specialization within generalist pollinator species has received little attention.

Generalist pollinator species may have an innate, or average, preference for some plant species over others (e.g., due to morphological adaptations such as tongue length (Harder, 1985) or balancing nutritional requirements (Williams & Tepedino, 2003)), but will nevertheless visit additional species depending on the foraging context (Bruninga‐Socolar et al., 2022). Generalist pollinator species may exhibit adaptive foraging in response to their environment, despite a general preference for common plant species, and therefore a segment of the pollinator community might visit rare plant species enough to reliably pollinate them (Benadi, 2015; Benadi et al., 2012; Bruninga‐Socolar et al., 2016, 2022; Goulson, 1994; Kunin & Iwasa, 1996; Revilla & Křivan, 2016; Valdovinos et al., 2013; Waser, 1978). Recent theoretical work on the role of pollination in plant species coexistence explores variation in pollinator species preferences and finds that the ability of generalist pollinator species to switch plant interaction partners and to specialize on different plant species promotes plant species coexistence (Benadi, 2015; Revilla & Křivan, 2016; Valdovinos et al., 2013).

For generalist pollinators, sequential visits to rare plant species may also result from variation between conspecific individuals in short‐term foraging behavior, that is, within individual foraging paths. Existing models of plant‐pollinator interactions and their effects on plant species coexistence assume that all pollinators or all pollinators of the same species forage identically (e.g., Benadi, 2015; Benadi et al., 2012, 2013; Essenberg, 2012; Levin & Anderson, 1970). Yet Heinrich (1976) qualitatively demonstrated that individual‐level specialization exists in bumblebees and recent work has shown small‐scale differences among individuals in the sequence of plants visited in foraging paths in the same experimental array (Saleh & Chittka, 2007). Individual‐level variation in foraging behavior should be especially important for species coexistence when a small number of species dominates the pollinator community (i.e., the species abundance distribution ubiquitous in ecological communities; McGill et al., 2007, Song & Feldman, 2014, Winfree et al., 2015). However, to our knowledge, very few studies have quantified variation among individual bees at the level of their foraging paths, where sequential visits to conspecific individuals of a rare plant species may occur. Song and Feldman (2014) use coupled plant‐pollinator population dynamics models to show that adaptive foraging among individuals of a single pollinator species promotes plant species coexistence. However, plant competition for resources other than pollinators has a stronger effect on plant coexistence than competition for pollinators in that study. Therefore, these results might fail to capture the dynamics of pollen‐limited systems where pollen limitation is the mechanism limiting plant population sizes. Further, while previous work shows interesting avenues for plant‐pollinator interactions to influence plant species coexistence, no study has modeled variation in the stepwise foraging decisions of individual bees (i.e., intraspecific variation among foraging paths), a key puzzle piece in understanding the dynamics of plant coexistence in pollen‐limited systems because of the requirement that pollen be delivered in sequential or nearly sequential visits for successful pollen transfer.

In this study, we focus on the potential contribution of individual‐level specialization to coexistence dynamics in a pollen‐limited two‐plant system. First, we use observed foraging paths of *Bombus terrestris* in experimental flower arrays to estimate the level of individual‐level specialization in a model generalist pollinator species. We then develop a simple, computationally efficient simulation model for the coexistence dynamics of two pollen‐limited plants competing for shared pollinators. We use this simulation to explore coexistence dynamics across a region of parameter space that spans variation in the average preferences of two pollinator species, variation in the magnitude of individual‐level variance around those averages, and variation in the starting abundances of the two plants. We ask whether and when realistic levels of individual variation in path‐level foraging preferences meaningfully alter coexistence dynamics. Finally, we review the sparse existing literature on the degree of individual specialization in nature and assess whether observed bee preferences are sufficient to support pollinator‐mediated plant coexistence.

## 2 MATERIALS AND METHODS

### 2.1 Experimental data on individual bee foraging

**FIGURE A1:**
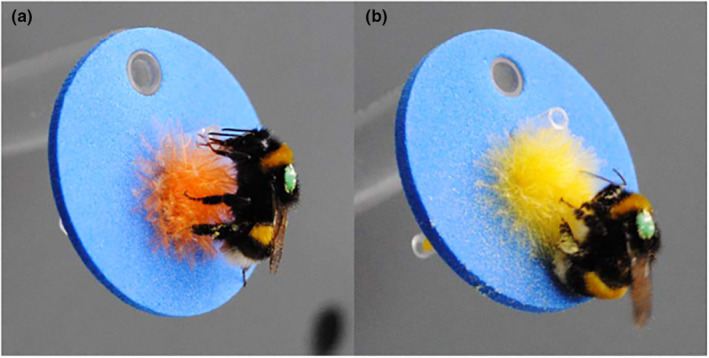
Artificial flowers containing pollen rewards (left) and nectar rewards (right). See Konzmann and Lunau (2014) for additional details.

Because individual variation in foraging paths is rarely measured empirically, we present experimental data that allows us to quantify variation in individual bee foraging behavior. We observed foraging bumblebees (*B. terrestris*) in a laboratory setting, using a repeated measures design to test for statistical differences in individual foraging behavior. Naïve, marked *B. terrestris* workers foraged in an artificial meadow with 42 artificial flowers consisting of blue discs with two types of floral guides: 21 flowers with yellow floral guides containing pollen rewards and 21 flowers with orange floral guides containing nectar rewards (Appendix 1: Figure A1). These two artificial flower types simulate two plant species from which bees get different rewards. We tested 41 *B. terrestris* workers in 199 individual trials across 20 combinations of pollen and nectar quantity and quality (Appendix 1: Table A1). Each worker was not tested for all combinations of rewards. Each worker completed a mean of 4.85 ± 0.72 trials, and a maximum of 16 trials. For each trial, we recorded the number of flowers visited of each type. To be counted as a visit, the bee had to directly contact and collect the floral reward. After each trial, we calculated the amount of reward collected by measuring the sugar solution left in the nectar flowers and weighing the pollen collected by the bee. See Konzmann and Lunau (2014) for additional details.

### 2.2 Data analysis

To assess whether individual bees differed in their foraging preferences, we modeled individual bee foraging decisions (probability of visiting pollen vs. nectar flowers) as a function of the quality and quantity of pollen and nectar rewards as well as a random effect of individual bee. If all bees are identical in preference, then the random effect variance should be indistinguishable from zero and including the random effect should not substantially improve the model fit. Therefore, we sought direct inference on the random effect variance to examine whether the data rule out near‐zero variance, where random effect variance indistinguishable from zero would indicate no differences between individual bees. Because frequentist model fits may underestimate the uncertainty in variance parameters (Kéry, 2010), we fit the model under a Bayesian mode of inference using vague priors and five chains, each with 1000 iterations of burn‐in and 50,000 iterations of sampling. The Gelman‐Rubin diagnostic was 1.0 for all margins, including the multivariate *Rhat* (“coda” package; Plummer et al., 2006). We compared prior and posterior distributions for the standard deviation to assess whether the data constrain the standard deviation away from zero. To confirm whether the random effect of individual variation should be in the best fit model, we compared the models with and without individual variation using both a frequentist likelihood ratio test and a Bayesian indicator variable analysis (Appendix 1). All analyses were done in the program R (R Core Team, 2017) using JAGS (Plummer, 2003) via the R package “rjags” (Plummer, 2016) and are publicly available (Bruninga‐Socolar et al., 2023).

### 2.3 Simulation model

We wish to investigate the dynamics of a plant‐pollinator community with two pollen‐limited annual plant species that are obligate on two shared pollinator species. To do so, we use a stochastic difference equation model to simulate the population size of both plant species through time under different bee foraging scenarios. This difference equation framework is well‐suited to model a plant‐pollinator system of annual plants and insects with annual life cycles because difference equations predict population size in each year based on what is known about the population in the previous year.

Our modeling framework simulates the number of plants of each species in each successive generation based on the number of successful pollen transfers and the probability that a fertilized ovule recruits to the adult stage in the next generation. For this study, we treat the latter probability as fixed because we are most interested in exploring parameters related to bee foraging. The model allows the number of successful pollen transfers to depend on bee foraging preferences, plant abundances, and the decay of pollen transfer after bees visit intervening flowers (Richards et al., 2009). We assume that pollinator populations are not limited by plant populations (e.g., they are nest site limited), such that the number of flower visits per year is constant. This assumption allows us to model the effects of a particular pollination regime on plant coexistence without modeling changes in the bee community over time. The extent to which bees are limited by non‐food resources such as nest sites is understudied (Harmon‐Threatt, 2020; Roulston & Goodell, 2011). However, a few studies document a positive relationship between nest site availability and bee abundance and species richness across nesting types (Potts et al., 2003, 2005) and demonstrate that neither floral nor nesting variables alone can explain bee population and community dynamics (Potts et al., 2005). For analytical simplicity and inferential clarity about pollinator‐mediated plant coexistence, we conceptualize our system as a nest site‐limited bee community. The model does not include a spatial component or other mechanisms of plant coexistence, such as competition for non‐pollinator resources.

We further assume that the identity of the plant in the *i*th visit delivered by the *j*th bee is independent of the plant identity on the previous visit and is conditioned on the bee's foraging preferences, described below. Thus, our simulation computes the probability that any single bee visit will deliver conspecific pollen, samples binomially from this probability and the total number of visits delivered by a given bee, and then sums across all bees in the community. Our full model is given by

(1) $$P_{1_{t+1}}\sim \text{binomial}\left(S_{1_{t}},\rho_{1}\right)$$

(2) $$S_{1_{t}}=\sum_{i}\sum_{j}V_{1_{\mathrm{ij}}}$$

(3) $$V_{1_{\mathrm{ij}}}\sim \text{binomial}\left(\nu_{\mathrm{ij}},\sum_{k}\tau_{k}{\left(\frac{{\left(\alpha_{\mathrm{ij}}P_{1_{t}}\right)}^{\beta }}{{\left(\alpha_{\mathrm{ij}}P_{1_{t}}\right)}^{\beta }+P_{2_{t}}^{\beta }}\right)}^{2}\right)$$

(4) $$P_{2_{t+1}}\sim \text{binomial}\left(S_{2_{t}},\rho_{2}\right)$$

(5) $$S_{2_{t}}=\sum_{i}\sum_{j}V_{2_{\mathrm{ij}}}$$

(6) $$V_{2_{\mathrm{ij}}}\sim \text{binomial}\left(\nu_{\mathrm{ij}},\sum_{k}\tau_{k}{\left(\frac{{\left(\alpha_{\mathrm{ij}}P_{1_{t}}\right)}^{\beta }}{{\left(\alpha_{\mathrm{ij}}P_{1_{t}}\right)}^{\beta }+P_{2_{t}}^{\beta }}\right)}^{2}\right)$$

where *P*_nt_ is the population size of plant species *n* in year t, *S*_nt_ is the number of successful pollen transfers to plant species *n* in year *t*, and *ρ*_n_ is the probability (treated as constant) that a fertilized ovule of plant species *n* recruits to an adult in the next generation (Table 1). *S*_nt_ is obtained by summing *V*_n_, the per‐bee number of successful pollen transfers to plant *n*, across all bee individuals *j* of all bee species *i*. To calculate *V*_nij_ for a given bee individual, we draw from a binomial distribution with number of trials *v*_ij_ equal to the total number of floral visits performed by the individual bee (treated as constant; Table 1). The per‐visit probability of successful pollen transfer is equal to the probability that sequential visits result in conspecific pollen transfer *τ*_k_ multiplied by the probability that visits are sequential. Note that *τ*_k_ is a vector whose first value is the probability of pollen transfer from immediately sequential visits, the second value is the probability of pollen transfer given one intervening visit to another plant species, and so on (Table 1). The values of *τ*_k_ are taken from Richards et al. (2009) who model variation in pollen deposition by individual pollinators. We assume that a visit that simultaneously delivers pollen from multiple previous visits to conspecific plants results in more total reproduction than a bee visit delivering pollen from fewer conspecifics.

**TABLE 1:** Ranges of parameter values in the simulation model.

Parameter	Value	Brief description	Values in sensitivity analyses
Bee species 1 mean preference, *μ*_1_	−10, −6, −4, −2, 0, 2, 4, 6, 10	Mean foraging preference of bee species 1. Together with the standard deviation of foraging preference of bee species 1, these values describe a lognormal distribution from which each individual bee's preference is sampled.	
Bee species 1 standard deviation, *σ*_1_	0, 1, 3		
Bee species 2 mean preference, *μ*_2_	0, 2, 4, 6, 10	Mean foraging preference of bee species 2. Together with the standard deviation of foraging preference of bee species 2, these values describe a lognormal distribution from which each individual bee's preference is sampled.	
Bee species 2 standard deviation, *σ*_1_	0, 1, 3		
Preference for rarity, *β*	0.1, 0.3, 0.5, 0.7, 0.9, 1.1, 1.3, 1.5, 1.7, 1.9	Assigned to each bee, this value represents the individual's preference for common (*β* > 1) versus rare (*β* < 1) plant species.	
Bee abundance, *A*_i_	100 per bee species (Appendix 1)		Both elevated: 150 bees (Appendix 1: Figure A2) Both lowered: 50 bees (Figure A3) Asymmetric elevated: 100 species 1, 150 species 2 (Figure A4) Asymmetric lowered: 100 species 1, 50 species 2 (Figure A5)
Initial plant population sizes	500,000 per plant species		
Pollen transfer probabilities, *τ*_k_	Sequentially for each visit in a bee foraging path: 1, 1, 1, 1, 0.7, 0.5, 0.2 (Richards et al., 2009)	A vector whose 1st value is the probability of conspecific pollen transfer from immediately sequential visits, the 2nd value is the probability given one intervening visit to another plant species, and so on.	Lowered: 0.7, 0.7, 0.7, 0.7, 0.49, 0.35, 0.14 (Figure A6) Faster decay: 1, 0.9, 0.7, 0.5, 0.3, 0.2, 0.1 (Figure A7)
Probability of pollen becoming an adult plant, *ρ*_n_	0.04 for both plant species (Appendix 1)	Probability that a fertilized ovule of plant species recruits to an adult in the next generation. Held constant across generations in our model.	Both elevated: 0.2 (Figure A8) Both lowered: 0.01 (Figure A9) Asymmetric elevated: 0.04 species 1, 0.05 species 2 (Figure A10) Asymmetric lowered: 0.04 species 1, 0.03 species 2 (Figure A11)
Visits per bee lifetime, *υ*_ij_	25,000 per individual for each bee species (Cane, 1997; Ribbands, 1949)	Total number of floral visits performed by each individual bee.	Both elevated: 30,000 visits (Figure A12) Both lowered: 20,000 visits (Figure A13)

*Note*: Bee species 2 is constrained to always have a positive mean preference because the system is symmetric with respect to bee species mean preferences; that is, the scenario where the bee species have opposite plant preferences (one has a positive mean and one has a negative mean) is captured by allowing bee species 1 to vary to negative values, and the situation where both species have negative mean preferences is the same as the situation where they both have positive mean preferences, except the identity of the mutually preferred plant species is switched. Parameter values are also provided for 12 model runs assessing the sensitivity of our results to the values related to the bee and plant populations.

The probability of sequential visits to plant *n* is a function of the foraging preference *α*_ij_ of the individual bee (constrained to take values greater than 0), the density of the plant species in year t, and a parameter *β* that controls whether the bee preferentially forages on common (*β* > 1) or rare (*β* < 1) plants. Generalist bees may forage preferentially on rare plant species to balance nutritional requirements, for example (Cook et al., 2003, Hendriksma & Shafir, 2016; see Section 4). *α*_ij_ and *β* thus modify the true density of each plant species to an effective density determined by each bee's species‐ and individual‐level preferences (*α* and *β*, respectively), and the probability that a given visit‐pair involves plant species *n* on both visits is the square of this effective density. Plant species 1 is preferred when *α*_ij_ is greater than 1, and plant species 2 is preferred when *α*_ij_ is less than 1. Each bee's *α* is sampled from a log‐normal distribution specified by its species mean preference and standard deviation, which represents the intraspecific variation in preference around the mean (Table 1):

(7) $$\alpha_{\mathrm{ij}}\sim \text{lognormal}\left(\mu_{i},\sigma_{i}\right),$$

where *μ*_i_ is the mean foraging preference of the *i*th bee species and σ_i_ is the standard deviation of the *i*th bee species. The mean and standard deviation are chosen a priori to span a wide range of possible values (Table 1). Values of *β* are systematically varied in the model to explore effects of preference for rarity on bee foraging and plant coexistence (Table 1).

**FIGURE A2:**
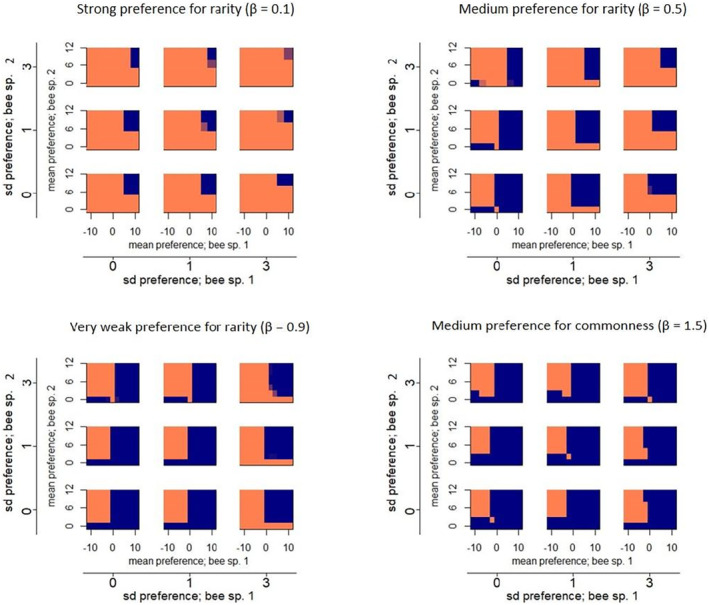
We ran the model 12 times to test the sensitivity of our results to the parameters listed in Table 1. Here, the values for bee abundance of each species are both elevated to 150 bees compared to abundances of 100 for our main model results (Figure 2). All other parameters in Table 1 stayed the same. All parameters given in Table 1 stayed the same.

**FIGURE A3:**
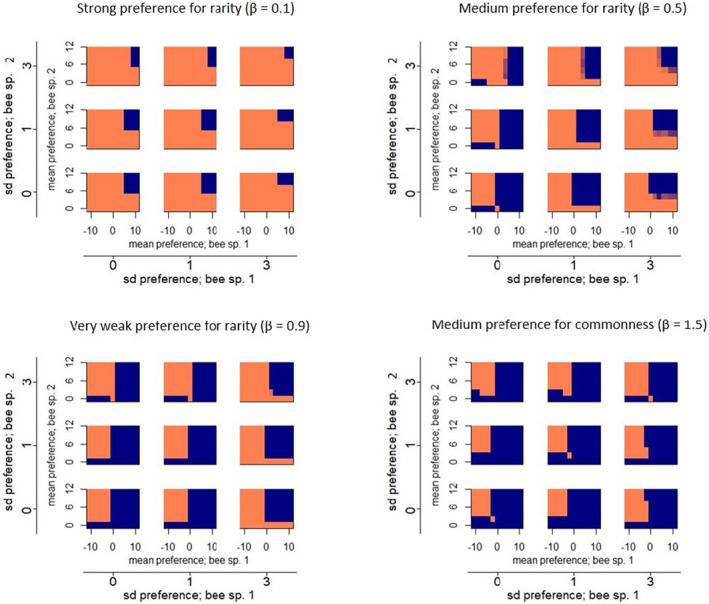
We ran the model 12 times to test the sensitivity of our results to the parameters listed in Table 1. Here, the values for bee abundance of each species are both lowered to 50 bees compared to abundances of 100 for our main model results (Figure 2). All other parameters in Table 1 stayed the same. All parameters given in Table 1 stayed the same.

**FIGURE A4:**
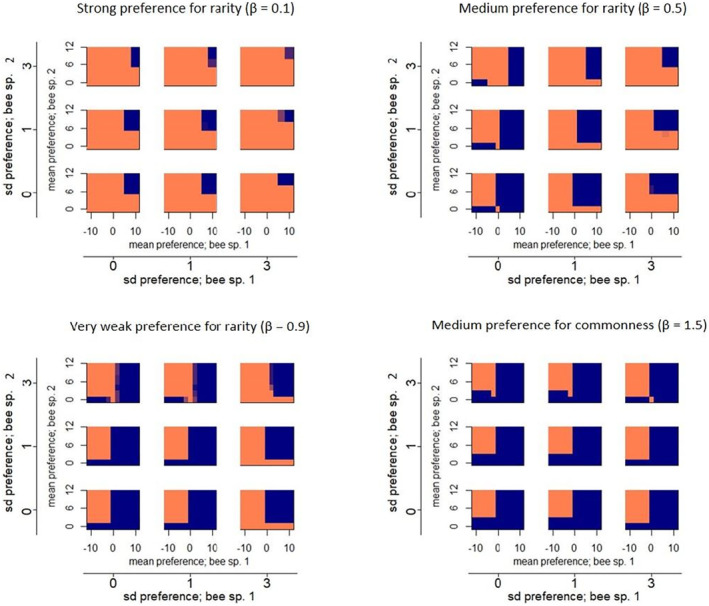
We ran the model 12 times to test the sensitivity of our results to the parameters listed in Table 1. Here, the values for bee abundance are asymmetric with species 1 set to 100 individuals and species 2 set to 150 individuals compared to abundances of 100 each for our main model results (Figure 2). All other parameters in Table 1 stayed the same. All parameters given in Table 1 stayed the same.

**FIGURE A5:**
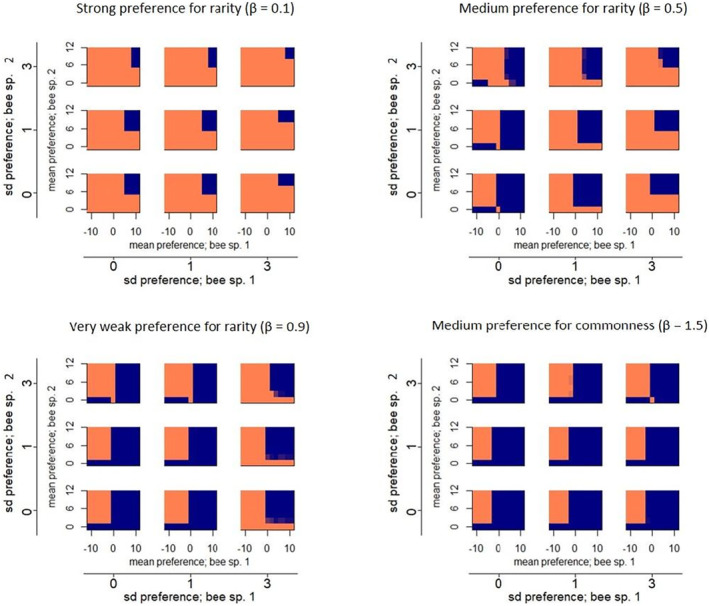
We ran the model 12 times to test the sensitivity of our results to the parameters listed in Table 1. Here, the values for bee abundance are asymmetric with species 1 set to 100 individuals and species 2 set to 50 individuals compared to abundances of 100 each for our main model results (Figure 2). All other parameters in Table 1 stayed the same. All parameters given in Table 1 stayed the same.

**FIGURE A6:**
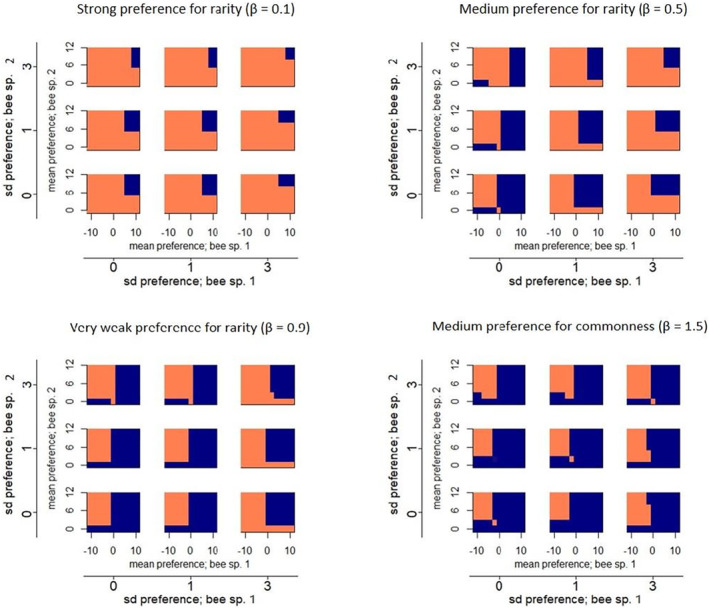
We ran the model 12 times to test the sensitivity of our results to the parameters listed in Table 1. Here, the values for the probability of conspecific pollen transfer are all lowered (0.7, 0.7, 0.7, 0.7, 0.49, 0.35, 0.14) compared to the set for our main model results (1, 1, 1, 1, 0.7, 0.5, 0.2; Figure 2). All other parameters in Table 1 stayed the same. All parameters given in Table 1 stayed the same.

**FIGURE A7:**
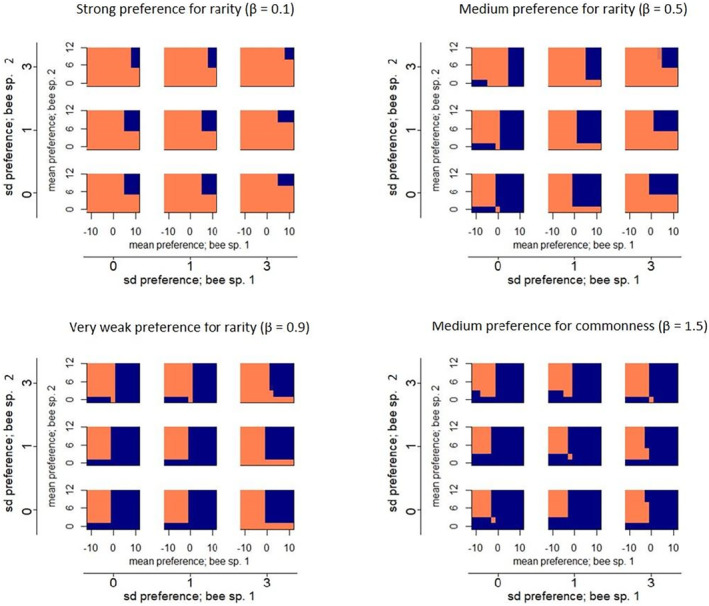
We ran the model 12 times to test the sensitivity of our results to the parameters listed in Table 1. Here, the values for the probability of conspecific pollen transfer decay faster (1, 0.9, 0.7, 0.5, 0.3, 0.2, 0.1) than the set for our main model results (1, 1, 1, 1, 0.7, 0.5, 0.2; Figure 2). All other parameters in Table 1 stayed the same. All parameters given in Table 1 stayed the same.

**FIGURE A8:**
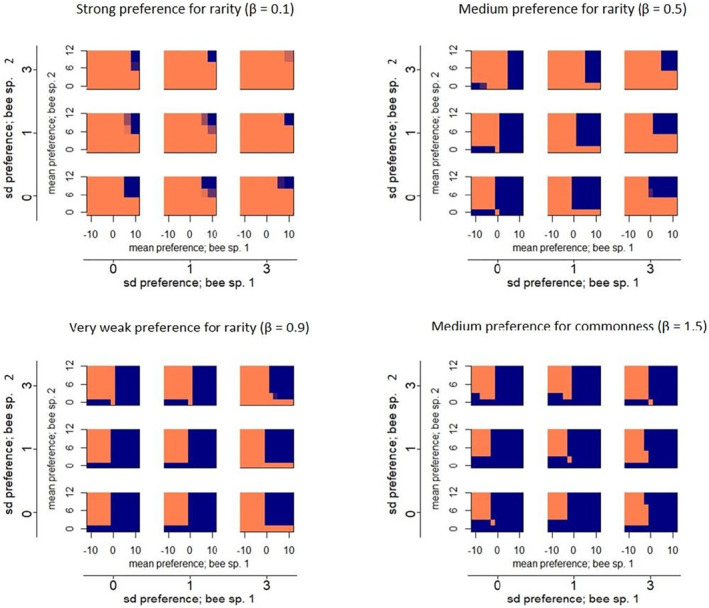
We ran the model 12 times to test the sensitivity of our results to the parameters listed in Table 1. Here, the probabilities of pollen becoming an adult plant of each species are both elevated to 0.2 compared to the value 0.04 used for our main model results (Figure 2). All other parameters in Table 1 stayed the same. All parameters given in Table 1 stayed the same.

**FIGURE A9:**
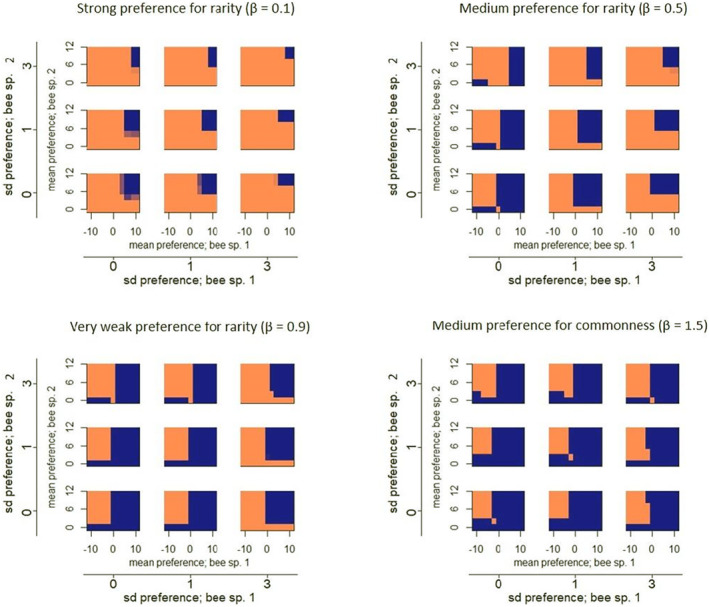
We ran the model 12 times to test the sensitivity of our results to the parameters listed in Table 1. Here, the probabilities of pollen becoming an adult plant of each species are both lowered to 0.01 compared to the value 0.04 used for our main model results (Figure 2). All other parameters in Table 1 stayed the same. All parameters given in Table 1 stayed the same.

**FIGURE A10:**
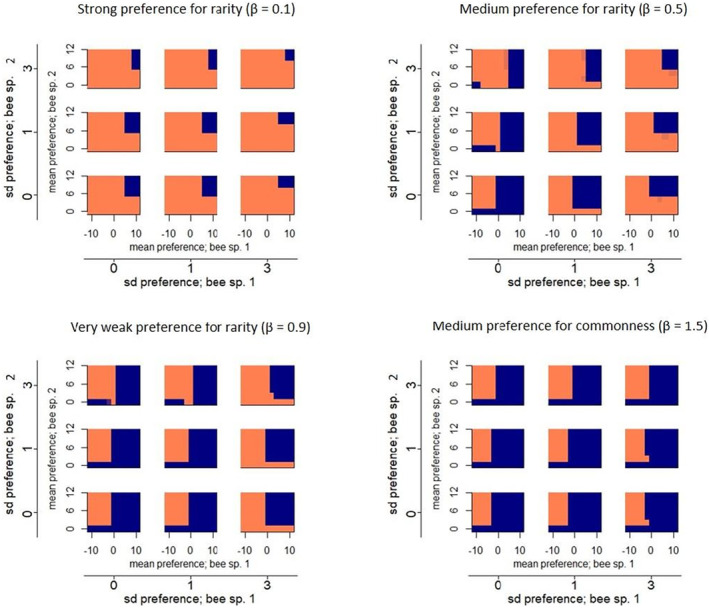
We ran the model 12 times to test the sensitivity of our results to the parameters listed in Table 1. Here, the probabilities of pollen becoming an adult plant of each species are asymmetric with species 1 set to 0.04 and species 2 set to 0.05 compared to 0.04 for both species for our main model results (Figure 2). All other parameters in Table 1 stayed the same. All parameters given in Table 1 stayed the same.

**FIGURE A11:**
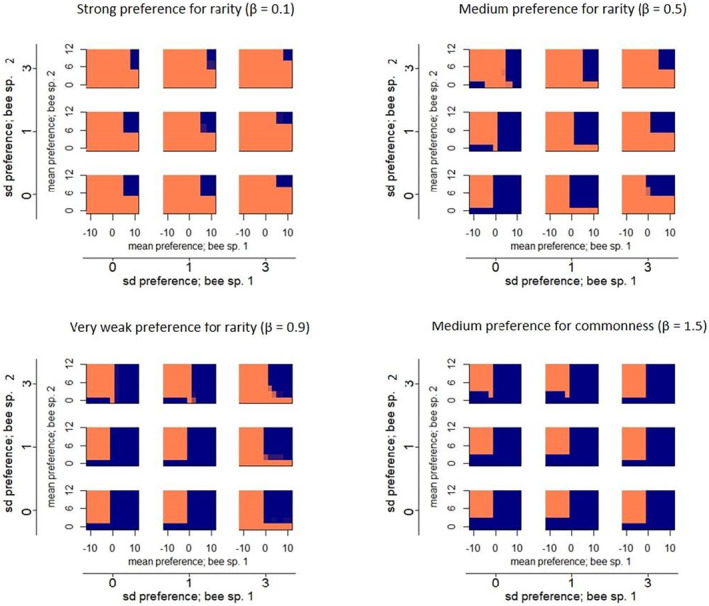
We ran the model 12 times to test the sensitivity of our results to the parameters listed in Table 1. Here, the probabilities of pollen becoming an adult plant of each species are asymmetric with species 1 set to 0.04 and species 2 set to 0.03 compared to 0.04 for both species for our main model results (Figure 2). All other parameters in Table 1 stayed the same. All parameters given in Table 1 stayed the same.

**FIGURE A12:**
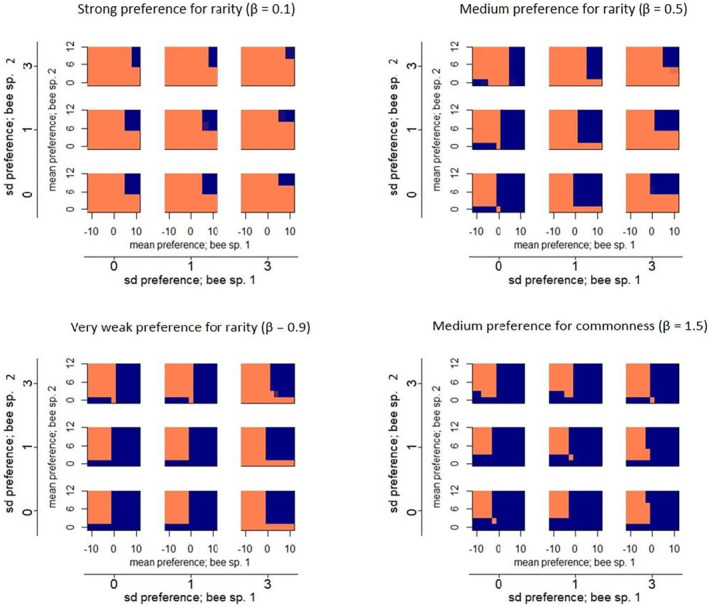
We ran the model 12 times to test the sensitivity of our results to the parameters listed in Table 1. Here, the lifetime visits per bee individual are set to 30,000 for each bee species compared to 25,000 used in our main model results (Figure 2). All other parameters in Table 1 stayed the same. All parameters given in Table 1 stayed the same.

**FIGURE A13:**
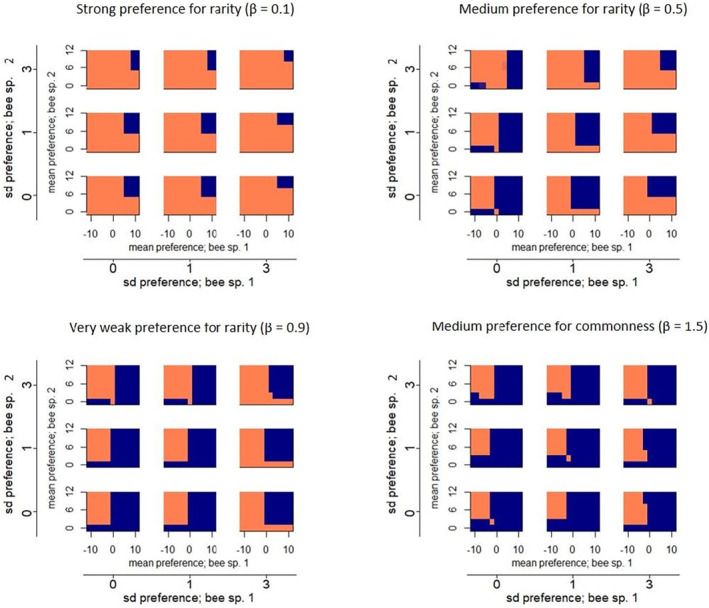
We ran the model 12 times to test the sensitivity of our results to the parameters listed in Table 1. Here, the lifetime visits per bee individual are set to 20,000 for each bee species compared to 25,000 used in our main model results (Figure 2). All other parameters in Table 1 stayed the same. All parameters given in Table 1 stayed the same.

All simulations were run in program R (R Core Team, 2017) for 100 plant generations and the R script is publicly available (Bruninga‐Socolar et al., 2023). In the infinite time limit, the only possible outcome of our simulation is eventual extinction of both plant species, but we found that all simulations rapidly settled into a well‐defined basin of attraction in many fewer than 100 iterations, and moreover that in all cases (for all choices of parameter values and starting conditions), 100 replicate simulations from identical starting conditions universally found the same basin of attraction and remained there after 100 iterations. We ran simulations to explore the range of parameter space of the bee species means, standard deviations, and preference for rarity to determine how bee preferences affect plant coexistence (Table 1). Parameters unrelated to bee foraging were held constant in most simulations (Table 1). We set the initial plant populations to very large values, which after the first timestep fall to numbers in line with what the pollinator population can sustain. In most simulations we initialized the plants at equal abundance (500,000 individuals each). We also explicitly investigated the ability of the pollinator community to support a rare plant species by systematically varying the initial abundance ratio of the two plants. The plant populations shared a constant population of 200 individual pollinators that each visited 25,000 plants per generation (Table 1: Appendix 1). Our choice of 200 pollinators was based on our empirical field experience in natural pollinator communities where floral resources are often patchily distributed (e.g., Bruninga‐Socolar et al., 2022), likely creating somewhat isolated but well‐mixed pollinator communities. In our models, 200 pollinators is enough to see stable, predictable dynamics, so there is no inferential gain in increasing the number. The number of plants visited per bee per generation was obtained from the literature (Cane, 1997; Ribbands, 1949). To determine whether our model results are sensitive to the values of bee abundance, pollen transfer probabilities, the probability of a fertilized ovule becoming an adult plant, or the number of visits per bee lifetime, we ran the entire simulation 12 additional times, varying one of these parameters in each model run (Table 1). These sensitivity analyses do not qualitatively change our results (Appendix 1: Figures A2, A3, A4, A5, A6, A7, A8, A9, A10, A11, A12, A13). We ran the model two additional times to determine the exact values of bee species mean preference required for coexistence for two specific cases, as described in the results: (1) where the plant species begin the simulation in equal abundance, and there is no intraspecific variation or preference for rare plants in bees, and (2) where the plant species begin the simulation with unequal abundance, and there is no intraspecific variation or preference for rare plants in bees.

### 2.4 Coexistence in our simulation context

Traditionally, ecological coexistence is analyzed based on the invasibility criterion: Can a population increase when rare (Chesson, 2000)? In pollinator‐mediated coexistence, this criterion is difficult to meet because sequential visitation rates to a rare plant species should approach zero in the limit of low relative abundance. Instead, we hypothesized that coexistence will often be stable within a limited basin of attraction at intermediate relative abundance. Therefore, we measure the strength of coexistence as the fraction of communities with both plant species persisting after 100 generations. For each combination of parameter values, we run 100 simulations to calculate this fraction. Thus, our criterion for coexistence is not invasibility, but rather the requirement that neither plant is likely to leave the basin of attraction and go extinct over our 100‐generation time window (Caswell, 1978; Socolar & Washburne, 2015; Valdovinos et al., 2013).

## 3 RESULTS

### 3.1 Experimental data on individual bee foraging

**FIGURE 1:**
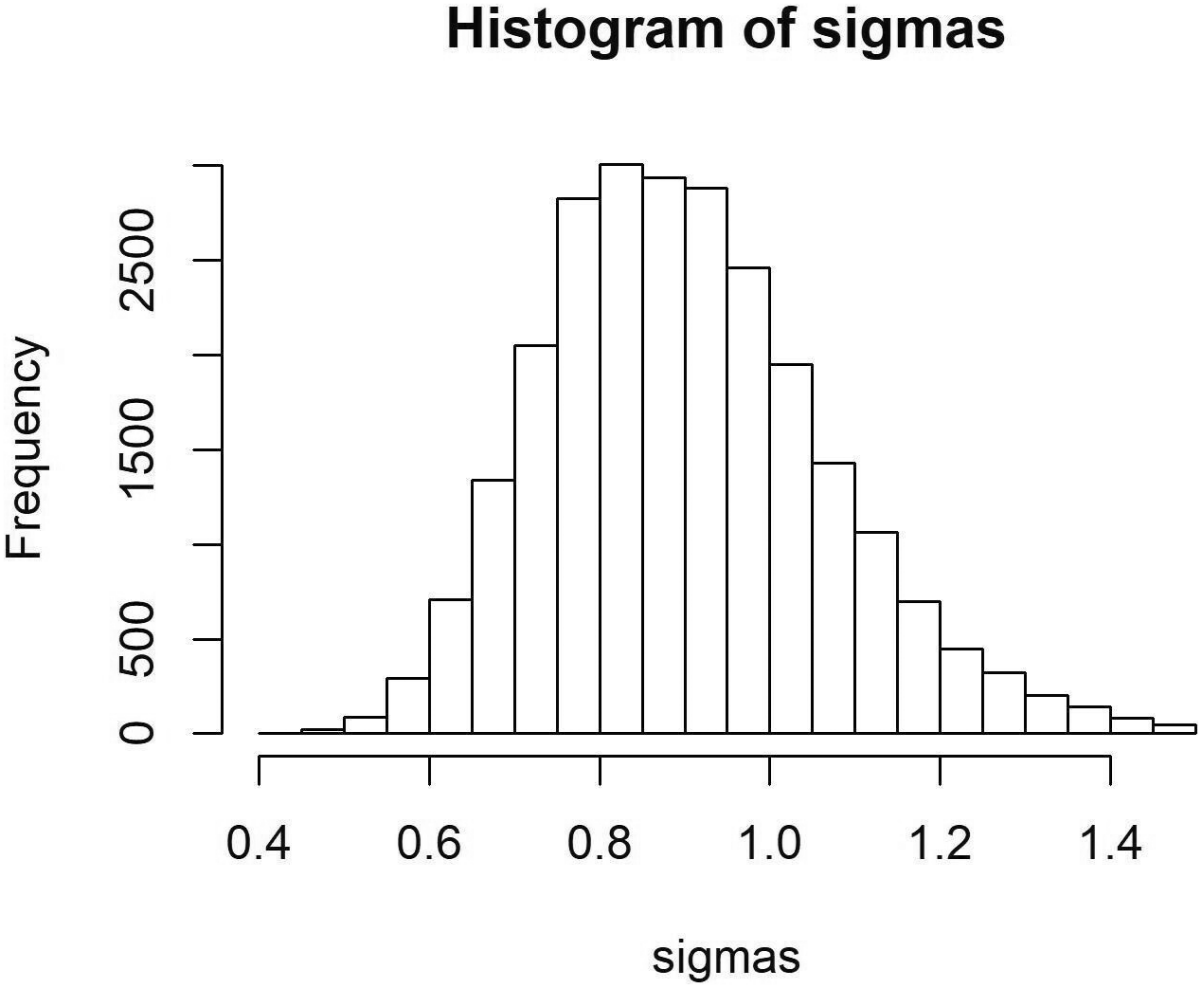
Posterior distribution of *σ*, the standard deviation of the random effect representing variation among individual bees in the model of bee foraging behavior. σ is constrained around 0.8–0.9 (95% credible interval of 0.62–1.27), indicating that the random effect variance is distinguishable from zero. If σ were constrained around zero, the model would not include variation among individual bees.

**FIGURE 2:**
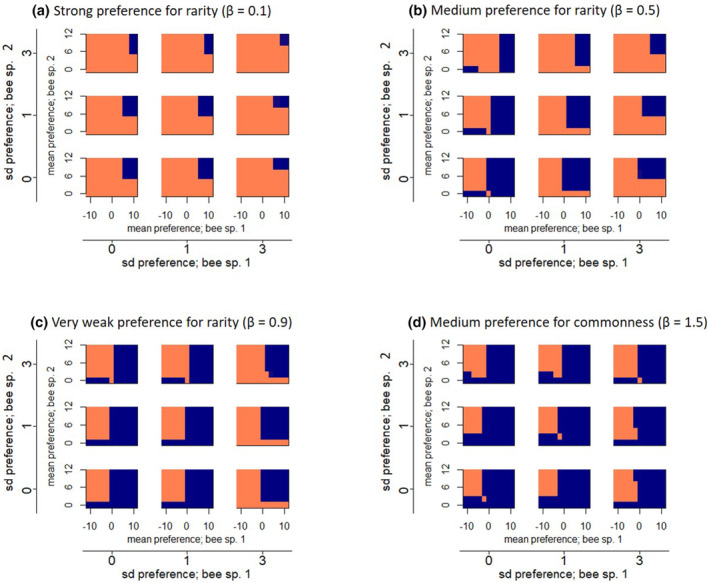
In all panels, the orange regions indicate plant species coexistence and the blue regions indicate exclusion of one species. The diagonal white line in each plot indicates the 1:1 relationship between positive mean bee preferences of bee species 1 and 2. This figure shows results from the case where the two plant species started at equal initial densities. With strong bee preference for rare plants (a), the two plant species coexist across most values of the bee species' mean preferences, where positive mean values indicate preference for plant species 1 and negative mean values indicate preference for plant species 2 (left half of every plot). The two plant species do not coexist where both bee species have a strong preference for the same plant species, indicated by high positive values of both means (right half of every plot). When the bee species prefer different plant species, as indicated by one positive mean and one negative mean, plant species coexistence is supported (left side of all plots). When the bee species' means are equal, there is no interspecific difference in preference. High standard deviation (high within‐species individual variation) around both bee species means increase coexistence. The greatest coexistence is obtained when there are high levels of variation around both bee species' means (high standard deviation; upper right plot of each panel has the greatest extent of orange compared to other plots within the same panel). When only one bee species' standard deviation is high, coexistence occurs less than in the previous case but more than when variation is low for both species (compare top row or right column of plots to bottom left plot of all panels). When bees prefer common plants (d), even high levels of intraspecific variation among individuals, as indicated by high values of each bee species' standard deviation, cannot substantially increase coexistence. Intermediate levels of bee preference for rare plants show intermediate results (b, c).

The posterior distribution of the standard deviation of the random effect was constrained away from zero (Figure 1), indicating that variation in the random effect, attributable to variation among individual bees, is included in the best model. The standard deviation of the random effect is mathematically equivalent to the standard deviation of bee species preferences in our simulation model. In our data analysis, the standard deviation (sigma or *σ*) takes a value close to 1. In our simulation model, increasing the standard deviation from 0 to 1 in agreement with our experimental result had a very minor effect on plant species coexistence when there was no preference for rare plants (Figure 2c), but improved coexistence slightly when there was moderate preference for rare plants (Figure 2b). When there is no interspecific variation (bee species' means are equal), intraspecific variation only improves coexistence when the standard deviation of at least one species is 3, a level much higher than our experimental result (Figure 2). The likelihood ratio test and Bayesian indicator variable analysis confirmed that individual variation is included in the best fit model (Appendix 1).

### 3.2 Effects of variation in bee foraging preferences on plant coexistence

Our simulation model of individual bee foragers requires strong bee specialization for plant coexistence. Bee specialization in the model is a result of density‐dependent preference for rare plants and/or strong variation in bee preference, such that different individuals strongly prefer different plant species. In our model, this variation could arise from differential species‐specific mean preferences (*μ*_i_), high levels of individual variation around the means (*σ*_i_), or differences among individual bees in their preference for common versus rare plants (*β*).

**FIGURE 3:**
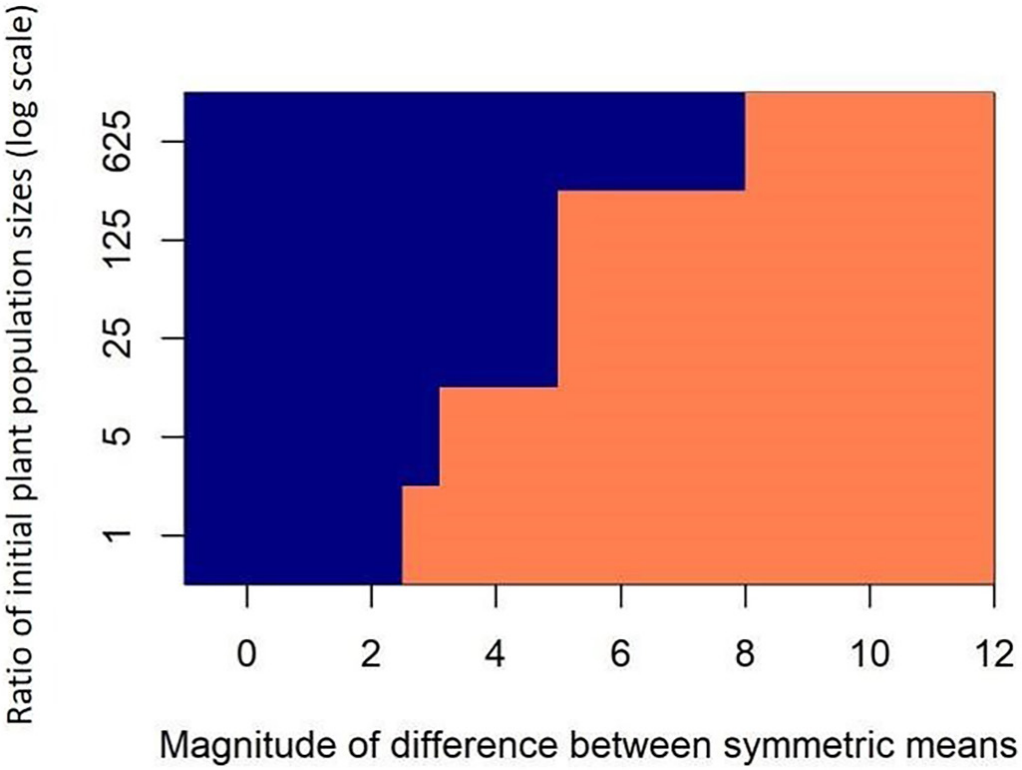
With no preference for rare versus common plants (*β* = 1) and no intraspecific bee variation (*σ* = 0), the two plant species coexist (orange regions of graph) with large differences between the mean bee species' preferences. Coexistence is more difficult as the ratio of the initial plant population sizes increases, that is, if one plant species starts out as rare compared to the second plant species. When the initial plant population sizes are equal, coexistence is obtained at smaller differences between the mean bee species' preferences.

Differences in the mean preferences of bee species have a large effect on plant coexistence in our model, that is, one bee species has a positive mean preference value and the other has a negative mean preference value, where positive values indicate preference for plant species 1 and negative values indicate preference for plant species 2 (Figure 2). Strongly opposing differences in mean preference always promote plant coexistence (Figure 2, left half of all plots), even when density‐dependent preference for rare plants is weak (Figure 2b) or non‐existent (Figure 2c,d), that is, pollinators prefer to visit common plants. When initial plant population sizes vary such that one plant species starts out as rare, stronger bee specialization (greater difference between μ_1_ and μ_2_) is required for coexistence in our model (Figure 3). With a strongly skewed ratio of initial plant population sizes (e.g., 625:1) extremely high levels of specialization are required for coexistence (Figure 3). When the initial plant population sizes are equal, smaller differences between the bee species' means maintain coexistence (Figure 3).

In our model, high standard deviation around the mean bee species preference allows individual foragers to differ from their species means. High standard deviation around the mean preference in one or both species increases coexistence slightly in our model communities (Figure 2a–d; compare bottom left plot of all panels to upper right plot of the same panel). Interestingly, the effect of intraspecific variation is more noticeable when there is medium preference for rare plants (Figure 2b; compare bottom left plot to upper right plot of the same panel).

When bees have a strong density‐dependent preference for rare plants, coexistence is achieved for all parameter combinations except where both bee species have a strong mean preference for the same plant species (Figure 2a; upper right corner of all plots). As density‐dependent preference for rare plants decreases (Figure 2b–d), fewer of the modeled plant communities coexist, although this decrease is attenuated by the effects of the bee species' mean preferences and the standard deviation around those preferences described above.

To provide a numerical example of the strength of pollinator specialization from our model, we calculated the percentage of visits to each plant species in a bee foraging path using the minimum parameter values required for coexistence. We assume the starting conditions of the model (Table 1; equal populations of the two plants), no variation around each bee species mean, and mean values of 1.3 and −1.3 for bee species 1 and 2, respectively (Figure 2c). An individual bee's preference is drawn from a log‐normal distribution with a mean of 1.3 or −1.3, depending on its species identity, and no standard deviation (Equation (7)). The resulting value from this distribution (3.67 from a mean of 1.3; 0.27 from a mean of −1.3) is α in Equation (3). For the purposes of this example, we assume that *β* = 1 (no preference for rarity) and the effective density of plant species 1 thus becomes (α*P_1t_)/((α*P_1t_) + *P*_2t_) (Equation (3)). For a bee species mean of 1.3, the effective density of plant species 1 is 0.79, an increase from its “true” density of 0.5. Thus, 79% of the individual bee's visits go to plant species 1. For a bee species mean of −1.3, 79% of the individual bee's visits go to plant species 2. Our model requires high levels of individual pollinator specialization within foraging paths for plant coexistence; that is, the vast majority of visits within a single foraging trip must be to a single plant species.

For the case where the initial population sizes of the plant species are unequal, for example with a ratio of 25:1, the bee species means must differ in magnitude by 4.4 for coexistence to occur, that is, the mean preference of bee species 1 is 2.2 and the mean preference of bee species 2 is −2.2 (Figure 3). Repeating our calculation above assuming that *β* = 1 and there is no intraspecific variation in bee foraging behavior, the α values for bee species 1 and bee species 2 are 9.03 and 0.11, respectively (Equation (7)). Using Equation (3), approximately 99.6% of the visits of an individual bee of species 1 go to plant species 1 (the common plant), and 0.04% of visits of an individual bee of species 1 go to plant species 2 (the rare plant). Approximately 73% of the visits of an individual bee of species 2 go to plant species 1, and 27% of visits of an individual bee of species 2 go to plant species 2. Thus, with mean preference values of 2.2 and −2.2 for bee species 1 and bee species 2, respectively, the rarer plant species 2 receives a non‐trivial percentage (27%) of visits from one of the bee species. When bee species have strongly diverging mean preferences such that one species prefers the rare plant, pollinator visits to the rare plant occur. If we use these same preference values (±2.2) to calculate bee preference when the plant species are in equal mixture for comparison to our first example, we find that 90% of visits in a foraging path of each bee species must go to their preferred plant species, compared to 79% with preference values of ±1.3.

## 4 DISCUSSION

Our simulations show that only highly specialized pollinators permit coexistence in pollen‐limited plant communities. Therefore, we predict that where they exist, pollen‐limited plant communities should interact with specialized pollinator communities, where specialization is quantifiable at the pollinator species level and/or the level of the individual forager. Our simulation model provides quantitative benchmarks for the within‐foraging‐bout visitation frequency to each plant species in order to support coexistence. To maintain coexistence of two plants with equal initial abundance, our model requires that at least some individual foraging paths must visit each plant species approximately 79% of the time. Preferences must be even stronger to buffer against unequal initial population sizes. For example, if plant species abundance ratios start at 25:1, coexistence emerges only when some bees have preferences of α equal to ±2.2 (Figure 3), which corresponds to delivering over 90% of visits to one plant when the plants are in equal mixture.

Our experimental results using bumblebees suggest that individual‐level specialization exists in foraging pollinators in the lab (i.e., intraspecific variation; Figure 1). However, the level of observed variation (SD = 1) does not appreciably affect plant coexistence in our model when there is no preference for rare plants (Figure 2c), but did improve coexistence slightly when there was moderate preference for rare plants (Figure 2b). Other studies have analyzed floral choice among bumblebee and honeybee workers, but did not calculate means of individual behavior for quantitative comparison (Grüter et al., 2011; Heinrich, 1976, 1979). While our experiment was simple and does not match real‐world conditions, existing literature on bee specialization within foraging paths suggests that specialization within paths may be high enough some of the time to promote plant coexistence (see below). Further work in natural systems is needed to quantify natural variation in individual bee foraging behavior and describe under what conditions such variation occurs. In particular, studies that record plant visit sequences within bee foraging paths and quantify the relative rarity versus commonness of available plant species are necessary (Thomson, 1981; Waser, 1986).

Very little previous work has investigated effects of specialization of individual foragers of a generalist pollinator species on plant species coexistence. Song and Feldman (2014) found that some pollinators specialize on the plant species that is rarer in a two‐species community only when its local density is high, which they suggest is more likely when total plant density in the community is high. We add that spatial clumping of conspecifics within a plant community could have a similar effect as bees forage optimally on plants that are close together. A recent empirical study suggests that bee responses to conspecific spatial clumping of plants in a multi‐species community may drive their stepwise foraging choices within foraging paths, affecting the delivery of sequential visits to the same plant species and resulting in persistence of rare species in the community (Bruninga‐Socolar et al., 2022). Most studies of the effects of pollinator visitation on plant species coexistence are conducted in a network context (e.g., Valdovinos et al., 2013) or using differential equations that model populations over time without modeling foraging paths (e.g., Revilla & Křivan, 2016). Both approaches elide the short‐term dynamics of effects of bee foraging behavior on pollen delivery to plants.

A key question is whether the levels of pollinator specialization that drive pollinator‐mediated coexistence in our model are widespread in nature. We consider four mechanisms that might deliver sufficiently specialized within‐bout foraging dynamics: species‐level specialization, individual‐ or bout‐level specialization, density‐dependent preference for rare plants, and spatial or temporal clumping of flowers.

### 4.1 Species‐level specialization

Most bees are not dietary specialists, and very few bee species are monolectic (specialized on the pollen of a single plant species) (Michener, 2000). However, oligolecty (specialization on a plant genus or family) is not uncommon (Michener, 2000). For example, 43% of bee species in the tribe Anthidiini are oligolectic at the level of plant family, subfamily, or tribe (Müller, 1996), and 30% of bee species in a region of subtropical Brazil are oligolectic (Schlindwein, 1998). Thus, in species‐poor systems with a single plant per family, oligolectic bees might provide a powerful mechanism for plant species coexistence if they behave as monolectic. Note that competition between two species of congeneric plants for oligolectic pollinators is precisely analogous to the two‐plant scenario for generalist pollinators in our model.

Documented cases of strongly monolectic pollinators are rare and often involve unusual examples of coevolution, such as the classic example of long‐tongued moths and long‐spurred orchids (e.g., Netz & Renner, 2017). In such specialized cases where bees and plants have a strong reciprocal preference, pollinators might easily mediate persistence of the plant in arbitrarily species‐rich systems, and the selective forces that guided the evolution of such elaborate signaling seem likely to involve pollen limitation (Kiester et al., 1984). However, we expect that these extraordinary cases account for the persistence of only a small minority of plant species.

### 4.2 Individual‐ or bout‐level specialization

Evidence of high individual bee specialization in nature is limited. While multiple studies report the frequency of visits to a given plant species within a foraging bout, these studies generally are not accompanied by data on the relative abundance of that plant within the community. Thus, it is possible (and in our view likely) that reported cases of high apparent specialization simply reflect preferential visitation to common plant species (or the commonest plant among the genus or family preferred by an oligolectic bee). Nevertheless, we note that many reported foraging paths are entirely restricted to a single plant species, which suggests that pollinator‐mediated coexistence might be possible. In an alpine system, 77% of individual bumblebees visited only one plant species within a foraging bout (Brosi & Briggs, 2013), and in an Australian garden, 88% of foraging trips of a stingless bee consisted of visits to only one plant species (White et al., 2001). However, only 35% of bumblebee foraging paths in a German meadow visited exclusively one plant species, and 37% of foraging paths included visits to at least three plant species (Raine & Chittka, 2007). In an agricultural system in Uruguay, approximately 80% of individuals of two bumblebee species collected only one type of pollen in corbicular pollen loads (Rossi et al., 2015). However, only 60% of individuals carried one type of pollen in nectar expressed from the abdomen, including most of the bees whose corbicular pollen loads were also tested (Rossi et al., 2015). Thus, studies that only examine corbicular pollen loads as a test of constancy may underestimate the diversity of plants visited within foraging paths. Among solitary bees, the species *Ceratina australensis* shows a high degree of specialization among individual bees as measured by comparing the composition of individual larval pollen provisions to mean population‐level pollen composition (Gaiarsa et al., 2022). Three species in the genus *Osmia* collected only one plant family in 44%–58% of pollen loads (each pollen load corresponds to one foraging path, subject to the caveat above; Eckhardt et al., 2014). However, two additional *Osmia* species showed no specialization within foraging paths at all (Cane, 2011). It is worth noting that nectar is a refillable resource in many plant species whereas pollen is not; therefore, individual bees foraging for pollen may be more likely to forage on rare plant species if the pollen resources offered by their preferred or common plant species have been exhausted.

One way to circumvent the need for data on the relative abundance of plants is to use Thomson's interview method of assessing bee preference, in which individual foragers are experimentally confronted with a choice of flowers of different plant species offered to the bees by the researcher (Thomson, 1981). Two studies use this method to document high specialization of bumblebee foragers on either of two congeneric plant species (Raine & Chittka, 2005; Wilson & Stine, 1996). In a mixed field of white and red clover (*Trifolium repens* L. and *T. pratense* L., respectively), Wilson and Stine (1996) show that 68% of *Bombus vagans* workers chose white clover when interviewed if the previous flower they had visited was also white clover. 88% of workers chose red clover when interviewed if the previous flower they had visited was also red clover (Wilson & Stine, 1996). Raine and Chittka (2005) calculate a bee species‐specific constancy index that compares the number of visits to the same plant species as the previous flower visited to the number of visits to a different plant species than the previous flower visited. They calculated constancy indices for three bumblebee species and found that the constancy indices for these species ranged from partial constancy to complete constancy (Raine & Chittka, 2005). These results suggest that bout‐level specialization in bumblebees might be sufficient to promote coexistence even of congeneric plants. The 79% sequential visitation rate required by our model sits between the 88% and 68% sequential visitation rates to red and white clover, respectively, documented by Wilson and Stine (1996).

### 4.3 Density‐dependent preference for rare plants

Density‐dependent preference for rare plants is under‐explored and further empirical work in natural systems is needed. We expect that bee preference for rare plant species may occur in nature, for example, when generalist bees require a certain resource that only a specific plant species can provide, and that plant species happens to be rare in the community (Williams & Tepedino, 2003). Visitation to rare plants may provide pollinators a competitive advantage against other pollinator species (Valdovinos et al., 2013). Rare plants may also offer respite to generalist pollinators from harmful secondary compounds present in common plants, in some systems (Bukovinsky et al., 2017; Eckhardt et al., 2014), or allow generalist pollinators to balance collection of multiple necessary nutrients, for example, essential amino acids (Cook et al., 2003; Hendriksma & Shafir, 2016). Bees are able to detect the nutritional properties of pollen both within and across plant species, suggesting that such fine‐scale adaptive foraging is possible (e.g., Ruedenauer et al., 2016, 2020; Vaudo et al., 2016). Indeed, several studies show that pollinator species exhibit temporal or spatial variation in foraging preference due to resource‐switching determined by plant species frequency (Bruninga‐Socolar et al., 2016; Campbell & Motten, 1985; Essenberg, 2012; Feldman, 2008; Ghazoul, 2006; Kunin, 1993, 1997b; Totland & Matthews, 1998; Valdovinos et al., 2013), but whether rare plants are visited sequentially within foraging paths has yet to be demonstrated empirically. In some systems, rare plants match the floral morphology and color of more common co‐occurring species (i.e., mimicry or signal standardization), suggesting that these plants do not rely on density‐dependent rare‐species advantage for pollination (De Camargo et al., 2019; Jersáková et al., 2016; Juillet et al., 2007; Lunau & Wester, 2017; Roy & Widmer, 1999).

### 4.4 Spatial or temporal clumping

Because of the high levels of individual bee specialization required by our simulation model for plant coexistence, we predict that in species‐rich, pollen‐limited plant communities, rare plant species should occur in clumps of high local abundance. Optimally foraging animals maximize energy gained per unit of time or effort (Schoener, 1971), or per nutrition type (e.g., amino versus fatty acids (Ruedenauer et al., 2021)). In the case of pollinators, transit between flowers is a key component of the time/effort denominator, and so spatial or temporal clumping of a rare plant species yields a higher probability of optimally foraging pollinators delivering sequential visits to conspecific plant individuals. If congeneric plants are clumped such that different species represent over 80% of flowering individuals in different spatial regions or time periods, then even neutrally foraging generalist pollinators could maintain coexistence and simultaneously reinforce the spatial clumping (if seed dispersal generally occurs over short distances) or temporal clumping. Interestingly, spatial clumping and/or mass flowering (temporal clumping) have been documented in plant species known to be pollen‐limited (e.g., Bruninga‐Socolar et al., 2022; Crone & Lesica, 2006) and recent work highlights that heterogeneity in floral resource availability affects pollinator visitation and plant reproduction (Labonté et al., 2023). More broadly, in many systems, certain plants or plant communities flower during short periods of the year, for example, spring ephemerals (Kudo et al., 2008).

## 5 CONCLUSIONS

The role of pollinators in mediating plant coexistence is of major interest both as a potentially important aspect of modern coexistence theory and for its basic and applied implications for pollen limitation: as a rule, diverse plant communities should not be severely pollen‐limited unless their pollinators tend to promote coexistence rather than exclusion. Previous work suggests that realistic levels of individual specialization can play a role in rescuing plant species coexistence when differences in mean species preferences—or overall density‐dependent preference for rarity—exists but is weak. Outside this regime, our results suggest that only extremely high levels of individual pollinator specialization are capable of maintaining coexistence in pollen‐limited plant communities. Species‐level specialization among bees is variable in nature, but may rarely be sufficient to promote coexistence among congeneric co‐flowering plants. Individual‐ and bout‐level foraging specialization, coupled with spatial clumping of rare plants, might be sufficient to provide a more general coexistence mechanism. We provide empirical evidence of intraspecific variation among bumblebee workers, but at a level insufficient to contribute strongly to plant coexistence in our simulation model. In the existing literature on bee foraging preference, we find that few studies permit rigorous quantification of bee foraging specialization, which requires quantitative data on the relative abundance of local plant species. However, a handful of studies using Thomson's interview method suggest that bumblebees might conceivably promote coexistence even among pollen‐limited congeners. We conclude that pollinator specialization should be included in models of plant coexistence and propose that future empirical work in pollen‐limited plant communities investigate the role of pollinator specialization in the persistence of those communities, particularly the persistence of rare plant species.

## AUTHOR CONTRIBUTIONS

**Bethanne Bruninga‐Socolar:** Conceptualization (lead); formal analysis (equal); writing – original draft (lead); writing – review and editing (equal). **Jacob B. Socolar:** Conceptualization (supporting); formal analysis (equal); writing – review and editing (equal). **Sabine Konzmann:** Formal analysis (equal); writing – review and editing (equal). **Klaus Lunau:** Conceptualization (supporting); formal analysis (equal); writing – review and editing (equal).

## Data Availability

The data and R code used in this paper were deposited in the figshare repository (Bruninga‐Socolar et al., 2023).

## APPENDIX 1

### Simulation model parameter choice

We selected biologically reasonable parameters based on the study system with which we are most familiar (Bruninga‐Socolar et al., 2016, 2022), where we estimate that roughly 200 bees may pollinate a patch of flowers and roughly 500,000 flowers may populate a patch. This field system differs from our simulation model system because the most abundant plant species is not annual. We determined the probability that a fertilized ovule recruits to the adult stage in the next generation based on the values we chose for the parameters in Table 1.

### Likelihood ratio test and indicator variable analysis

When comparing a random effects model with one without the random effect, standard AIC‐based approaches to model selection are difficult to implement because estimation of the number of effective degrees of freedom associated with the random effect is challenging (Kéry, 2010). Instead, we applied two alternative approaches to assess the strength of evidence for the inclusion of the random effect. First, we fit models with and without the random effect in the R package lme4, and we compared their fits using a likelihood ratio test. The likelihood ratio test between the models with and without the random effect representing variation among individual bees was significant (*χ* ^2^ = 125.67, *p* < .0001).

Second, we performed fully parametric Bayesian model selection using indicator variables (Hooten & Hobbs, 2015) to perform direct inference on whether the random effect should be included in the model. To do so, we modified the random effects model by multiplying the random effect variance by a Bernoulli indicator variable. When the indicator variable takes value zero, the random effect is excluded from the model, and when the variable takes value one, the random effect is included. The posterior distribution of the indicator variable is directly interpretable as the Bayesian posterior probability that the model including the random effect is the correct model. Our indicator variable analysis confirmed that the random effect is included in the best‐fit model (indicator variable *V* = 1 in 25,000 model runs). The Gelman‐Rubin diagnostic (multivariate *Rhat*) was 1.01.

**TABLE A1:** Combinations of rewards used in 20 tests of *Bombus terrestris* foraging behavior.

	Varied reward	Standardized reward
Nectar quantity	210, 105, 52.5, 21, 0 μL sugar solution (45%)	1 mL 100% pollen
Nectar quality	60%, 45%, 30%, 15%, 0% sugar solution (210 μL)	1 mL 100% pollen
Pollen quantity	1, 0.5, 0.25, 0.1, 0 mL pollen (100%)	210 μL 45% sugar solution
Pollen quality	100%, 75%, 50%, 25%, 0% pollen (1 mL)	210 μL 45% sugar solution

*Note*: For example, in each of five tests, bees were presented with a different value for nectar quantity while nectar quality, pollen quantity, and pollen quality were held constant. In the next series of five tests, bees were presented with a different value for nectar quality while nectar quantity, pollen quantity, and pollen quality were held constant, and so on for all 20 tests. See Konzmann and Lunau (2014) for additional details.
